# Septic Shock and the Aging Process: A Molecular Comparison

**DOI:** 10.3389/fimmu.2017.01389

**Published:** 2017-10-25

**Authors:** Fabiano Pinheiro da Silva, Marcel Cerqueira César Machado

**Affiliations:** ^1^Laboratório de Emergências Clínicas, Faculdade de Medicina FMUSP, Universidade de São Paulo, São Paulo, Brazil

**Keywords:** aging, systemic inflammation, immunity, sepsis, critical care

## Abstract

Aging is a continuous process promoted by both intrinsic and extrinsic factors that each trigger a multitude of molecular events. Increasing evidence supports a central role for inflammation in this progression. Here, we discuss how the low-grade chronic inflammation that characterizes aging is tightly interconnected with other important aspects of this process, such as DNA damage, mitochondrial dysfunction, and epigenetic changes. Similarly, inflammation also plays a critical role in many morbid conditions that affect patients who are admitted to Intensive Care. Although the inflammatory response is low grade and persistent in healthy aging while it is acute and severe in critically ill states, we hypothesize that both situations have important interconnections. Here, we performed an extensive review of the literature to investigate this potential link. Because sepsis is the most extensively studied disease and is the leading cause of death in Critical Care, we focus our discussion on comparing the inflammatory profile of healthy older people with that of patients in septic shock to explain why we believe that both situations have synergistic effects, leading to critically ill aged patients having a worse prognosis when compared with critically ill young patients.

## Introduction

Over time, improved health conditions have led to a steady growth in the older population, resulting in a substantial increase in the number of critically ill-aged patients. In addition, advanced age is associated with a worse outcome in all of the most frequent critical care conditions (1).

Chronic, low-grade, systemic inflammation, and the deregulation of several innate and acquired immune responses have been reported in seniors (2). The signaling pathways implicated in this scenario, called inflammaging, create a complex network (3–7) that is probably triggered and perpetuated by prolonged exposure to varied exogenous and endogenous factors, such as infection, tissue injury, DNA damage, mitochondrial dysfunction, intestinal barrier failure, and dysbiosis (8–10) and may contribute to the increased risk of acute illnesses, disability, and death in this population (11). Indeed, inappropriate inflammation and metabolic stress lead to the accumulation of senescent cells, which are characterized by transcriptional and epigenetic alterations that determine cell cycle arrest, as well as aberrant mRNA production and maturation, chromatin structure changes, and impaired proteostasis (12, 13). Moreover, emergency myelopoiesis and the persistence of immature myeloid cell progenitors have been shown to be significant contributors to dysfunctional inflammation in sepsis and are emerging in aging research (14, 15).

Pathogen-associated molecular patterns are molecules shared by many microorganisms, but not found in mammals, that are recognized by the immune system and activate cell defense. Several host factors are also able to alarm the immune system, even in sterile conditions. Indeed, persistent or recurrent contact with microbes (16), as well as with non-infectious danger signals, induce the production of damage-associated molecular patterns (DAMPs), which accumulate during aging (17). Examples include adenosine triphosphate, high mobility group box 1 protein, oxidized lipoproteins, heat shock proteins, and urate and cholesterol crystals. These DAMPs are able to activate membrane receptors and cytosolic receptors (including inflammasomes) or act directly at the nuclear level, inducing gene transcription (16, 18). Moreover, accumulating evidence suggests that mitochondrial and genomic DNA and histones also activate danger signals and induce systemic protection (19). For example, levels of circulating cell-free DNA, presumably released from damaged or dying cells, are increased in older adults (20) and are associated with both mortality and the magnitude of the inflammatory response (21–23). Similarly, critical inflammatory conditions, such as sepsis, are also characterized by high levels of circulating host DNA (24, 25).

Many nucleic acid molecular sensors have been found (26). CpG-enriched DNA, such as mitochondrial and bacterial DNA, are mostly recognized by TLR9 (21, 27), but other systems to detect not only mitochondrial and microbial DNA but also the nuclear DNA that migrates to the cytosol under pathologic conditions have been described (28–31). Furthermore, inside the nucleus, a sophisticated system of DNA sensors is able to detect DNA damage and activate immune signaling (31). In parallel, harmful DNA coming from pathogens, apoptotic cells, or DNA replication byproducts can be directly degraded by DNases to avoid the excessive activation of immune cascades (31).

### Enduring Permanent Aggression: From Mitochondrial Dysfunction to Genomic Instability

Aging is accompanied by a decline in mitochondrial function in all tissues; however, some tissues, such as the muscles, are particularly affected (32). Beyond their function in bioenergetics, growing research suggests that mitochondria participate in many other mechanisms that are deregulated in senescence (33). Indeed, mitochondria are important organelles in the maintenance of stem cells (12), the activation of the unfolded protein response (34), the regulation of innate and adaptive immune pathways (35, 36), and the modulation of the metabolic profile of the cell (32). As such, mitochondria are deeply integrated into cellular homeostasis (37).

Similarly, mitochondrial dysfunction is a common finding in a wide range of patients in critical care conditions (38–41). Pro-inflammatory mediators and oxidative stress impair the function of the respiratory chain enzyme complexes and damage the mitochondrial structure, including their genes (42, 43). While the genomic DNA can be affected in a similar manner, the mitochondrial DNA is likely more vulnerable to this type of damage due to its close location to the electron transport chain, its lack of protective histones, the limited efficiency of the mtDNA repair mechanisms, and the fact that, like bacterial DNA, it exclusively contains coding regions (44, 45). However, a single-cell contains thousands of mitochondrial genomes, and mutations of all of them in the same gene are unlikely, putting the nuclear genome at a higher risk (46). An excellent publication from Patananan et al. describes the current challenges to therapeutically modifying the mitochondrial genome and the important concept of heteroplasmy (47). Furthermore, emerging evidence suggests that DNA damage activates signaling from the nucleus to the mitochondria, creating complex networks that are crucial for mitochondria maintenance (48).

The genetic lesions arising from DNA damage include point mutations, translocations, gains, losses, strand breaks, and telomere shortening. DNA lesions occur frequently, even under physiological conditions (49). These genetic changes have a well-established impact on the aging process and have been largely investigated as both a cause and consequence of chronic low-grade inflammation. The same process may occur in the critically ill; however, it seems to occur over a short-time period and be massive in this population, while it is low-grade and persistent during aging.

The occurrence of DNA damage induces further immune activation, promoting a vicious circle with disastrous consequences (50). Indeed, there is increasing evidence pointing to reciprocal interactions between DNA damage, DNA repair, and the immune system (51). In the short term, depending on the intensity of damage or the presence of deficient DNA repair responses (52–54), genotoxic stress has the potential to induce aberrant cell responses, apoptosis, organ failure, and immunosuppression (19, 43). The occurrence and magnitude of acute DNA lesions in the setting of severe systemic inflammation, however, remain to be confirmed. In the long term, these plausible DNA mutations and deletions can significantly impact patient quality of life and predispose the survivors of inflammatory catastrophes to several morbid consequences. In this regard, a recent publication from our group showed that sepsis induces telomere shortening (55), confirming that inflammation affects telomere length (56) and that stress-induced premature senescence is a telomere-dependent process (57). Despite that, however, the other evidence in the literature addressing this topic is controversial and/or indirect. The phenotype of sepsis survivors, for example, resembles accelerated aging, and sepsis survivors suffer from a higher risk of additional morbidities, such as cardiovascular disease, cognitive impairment, tumor progression, and possibly death, for years following the sepsis event (58–60).

Since genomic instability is a hallmark of aging, genetic damage secondary to severe infection or other causes of critical illnesses may have a larger impact in seniors than in young patients. We believe that this is a critical factor that partially explains the worse outcome of older people, compared with the young, when affected by overwhelming inflammatory syndromes.

Redox reactions generate oxidatively modified signaling biomolecules that are crucial for the generation of appropriate innate and adaptive cell responses (35) and for many other fundamental biological processes (61–63). Reactive oxygen and nitrogen species have important physiological effects and display various well-established specific signaling functions, but they need to be tightly regulated; otherwise, they can generate significant tissue damage (64). Oxidative damage occurs to a larger extent both in older people and in the presence of acute or chronic inflammatory processes. It can lead to substantial DNA damage and significantly contribute to genomic instability and mitochondrial dysfunction. A DNA microarray study performed by our group found that the oxidative phosphorylation and the mitochondrial dysfunction pathways were the most-enriched pathways in septic patients of advanced age when compared with the young septic group (65).

### The Epigenetic Code: An Additional Layer of Complexity

Epigenetic changes involve various histone marks, DNA methylation, nucleosome positioning, and mechanisms governed by non-coding RNAs that are able to repress or activate transcription (66). Epigenetic alterations are important aspects in the regulation of aging, linking environmental factors with the genetic profile (67). We believe epigenetic modifications may be implicated in the global modifications to the cell response that manifest during the evolution of catastrophic inflammatory processes, especially those caused by overwhelming infection. In support of this hypothesis, a recent publication reported that sepsis in humans induces selective and precise chromatin modifications in distinct promoter regions of immunologically relevant genes (68). Cellular dysfunction secondary to genetic and epigenetic changes in the course of major inflammatory syndromes may be stochastic and partially reversible, even though some organs and tissues appear to be more strongly affected. Moreover, DNA regions that are robustly activated are likely more influenced since they are less protected than the heterochromatin. Unfortunately, this topic remains obscure in the critically ill. However, it could potentially explain, for example, the long-term cognitive impairment that is frequently detected in survivors of septic shock and other intriguing findings, such as the phenomenon of endotoxin tolerance (69).

There is a significant interconnection between the DNA damage response and epigenetic changes. DNA damage is a serious threat to cell viability, compromising the integrity of both the genome and the epigenome. The DNA damage response can lead to significant alterations in chromatin structure, affecting chromatin components and epigenetic marks, with major implications for cell metabolism (70). As stated by López-León and Goya, “aging seems to be characterized by a progressive depression of the transcriptional activity of chromatin” (71); this has been partially attributed to a reduction in DNA methylation and an altered chromatin architecture (72). The end result of epigenetic changes is aberrant gene expression, reactivation of transposable elements, and genomic instability (73).

There is increasing evidence that together with the above discussed factors, microRNAs and long non-coding RNAs also play a key role in fundamental epigenetic processes, with important implications for the aging process and various morbid states (74–76).

### Aging and Critical Illnesses: From Low-grade to Explosive Inflammation

The treatment of critically ill aged patients is challenging. Older people frequently exhibit atypical symptomatology, due to comorbidities and dysfunctions throughout all body systems that are related to the aging process (77).

Sepsis is a disease of the elderly. The incidence of sepsis increases exponentially with age, and sepsis-associated long-term sequelae particularly affect older patients. Sepsis survivors are at substantial risk for poor quality of life, functional disability, and cognitive impairment. As advances in medicine and quality of life extend the life expectancy worldwide, a growing number of aged patients need critical care (78). A recent study demonstrated a significant rise in survivorship after sepsis in the United States, caused by a rising incidence of sepsis rather than improvements in its case fatality rate, generating a substantial population burden of aged patients with disabilities (79).

**Figure 1:**
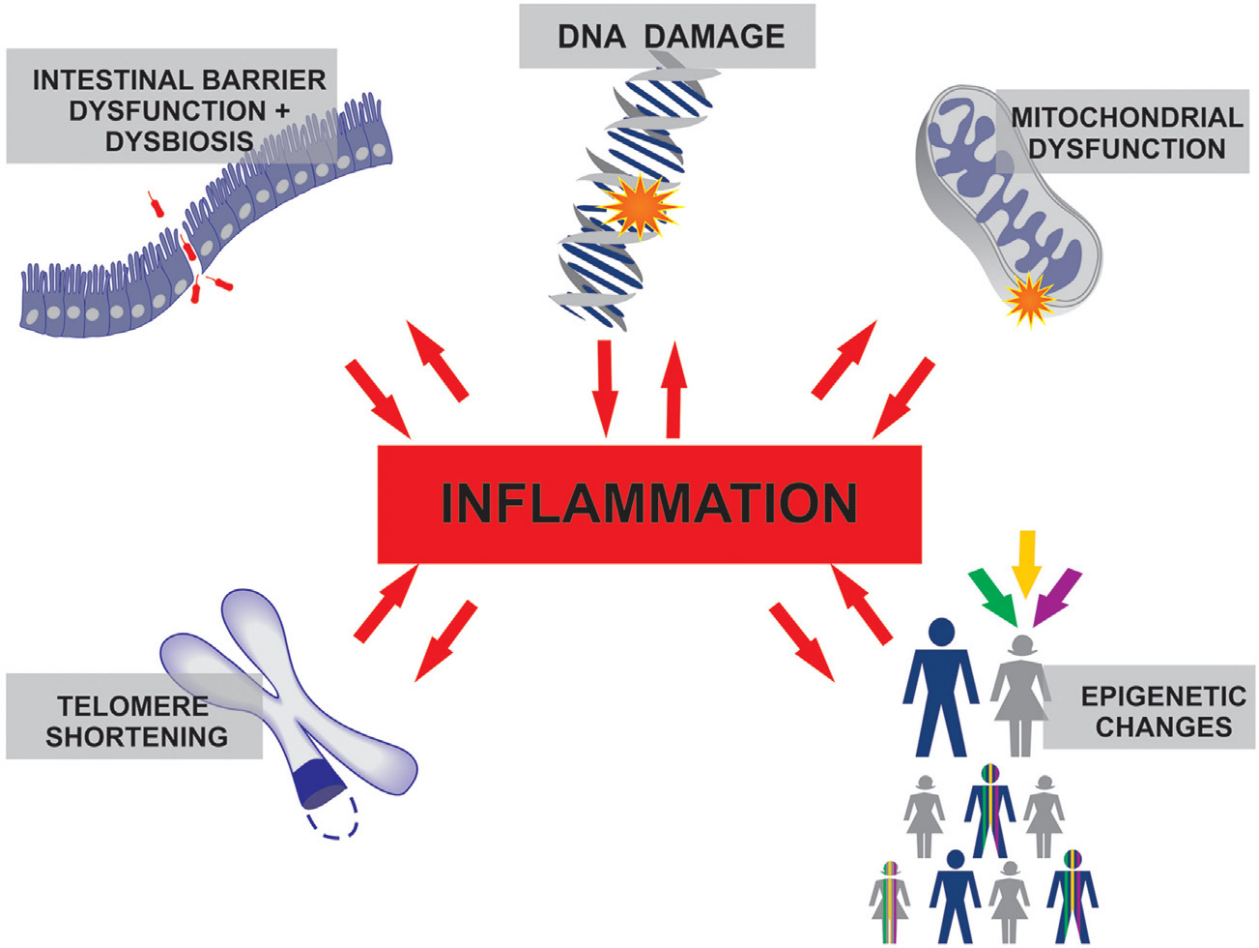
The inflammation that characterizes healthy aging and the inflammatory processes in several critical care conditions differ in duration and intensity, but both involve similar molecular interconnections where inflammation plays a central role.

The reason for the higher susceptibility to infection and increased mortality in older adults remains in debate (80). The basal inflammatory state found in healthy seniors suggests that aged people possess a limited capacity to control inflammation. Similarly, the critically ill are frequently affected by overwhelming inflammatory syndromes, where the host response is the major cause of damage. Examples include diseases such as septic shock, severe acute pancreatitis, burns, trauma, ischemia reperfusion injuries, and hemorrhages. As discussed earlier, the chronic low-grade inflammation in the elderly and the explosive inflammation in the critically ill share several commonalities. We propose that, together, these processes may have synergistic effects, leading to a worse outcome (Figure 1).

Notably, these synergistic effects have interesting peculiarities. A study performed by our group found that older people are as immunocompetent as young individuals regarding the cytokines, chemokines, and growth factors produced in response to devastating infections. After our analysis of several inflammatory mediators in the plasma of critically ill individuals, we were unable to find any reason that could serve to better explain why the aged show an increased susceptibility and mortality to septic shock (81). As detailed in the section below, this phenomenon can be partially explained by the fact that aged people probably display a prolonged inflammatory systemic response under acute stress conditions, when compared with the systemic response of the young, even though both groups share the same ability to trigger and sustain the same intensity of inflammatory signaling in the acute phase (81). Moreover, in a study performed in rats, we were able to demonstrate that despite a similar systemic response, aged rats show increased intestinal gene expression levels of TNFα, α-defensin 5, and α-defensin 7, when compared with young rats (82). Similarly, other work from our group demonstrated greater gene expression levels of COX-2 and intercellular junction proteins in the guts of aged rats with acute pancreatitis when compared with young rats in the same conditions, suggesting that, in situations of intestinal damage, the young animals are better able to restore intestinal barrier integrity (83). The results of these studies strongly suggest that the inflammatory response of the elderly is compartmentalized, with significant differences in the inflammatory profile depending on the organ under investigation.

For many years, the catastrophic systemic inflammation associated with many critical care diseases has been attributed to a massive and transient activation of the innate immune system, followed by a period of immunosuppression (84, 85). Seminal high-throughput gene expression studies performed in septic patients by the Wong group, however, challenge this theory. Indeed, instead of the classical biphasic curve, they have consistently detected an elliptical curve, formed by the persistent activation of innate immune genes in conjunction with widespread repression of gene programs corresponding to the adaptive immune system (86–88). Confirming these findings, a similar pattern was found in trauma patients (89), suggesting that infectious and non-infectious systemic inflammation in the critically ill may involve analogous cell responses. Notably, more recent studies are finding subtle differences in the transcriptional program of different acute stress conditions and even in different subsets of the same morbid process (90).

### Maintenance of the Intestinal Epithelial Barrier and the Human Microbiome

The intestinal mucosal barrier is a fundamental line of defense against undesirable luminal contents, such as microorganisms, toxins, and antigens, preventing their entrance into the bloodstream. It is mostly composed of epithelial cells, immune components, and mucus. Some researchers also consider the microbiome as part of the intestinal barrier (9, 91), since it helps to maintain the integrity of the intestinal barrier, providing nutrients and protecting against pathogens.

Aged people are in a persistent systemic inflammatory state that may be partially attributed to increased bacterial translocation, secondary to intestinal barrier dysfunction (92). As people age, the intestinal barrier weakens, partially due to decreased levels of tight junctions connecting epithelial cells, and the enteric immune system becomes ineffective (93). Indeed, higher plasma levels of lipopolysaccharide can be detected in the blood of older subjects, when compared with young individuals (94, 95). Furthermore, there is a shift in the intestinal microbiome after the age of 65, with an increased abundance of Bacteroidetes phyla (96) and a reduction in the capacity of the microbiota to carry out metabolic processes, such as short-chain fatty acid production (97). We propose that these previous alterations to the intestinal barrier in the elderly are probably exacerbated during systemic inflammation processes. In support of this idea, Zhang et al. recently demonstrated that neutrophil activation and aging are both affected by the intestinal microbiota and that depletion of the microbiome with broad-spectrum antibiotics significantly reduces the number of circulating aged neutrophils, ameliorating inflammation-related organ damage in a model of endotoxin shock (98). Another recent publication showed that germ-free mice do not display the increase in circulating cytokines that is a hallmark of aging and that co-housing germ-free mice with old, but not young, conventionally raised mice reconstitutes this phenotype; the authors concluded that, in mice, intestinal permeability increases with age due to microbial dysbiosis (99). Taken together, these observations suggest that the increased mortality of aged patients in critical care conditions is probably due to a prolonged systemic inflammatory response, at least partially caused by increased bacterial translocation and defective bacterial clearance (100).

Microbiome studies are challenging because there is extensive inter-individual variability, even among healthy subjects. Genetic, lifestyle, and environmental factors, such as diet, physical activity, geography, and exposure to xenobiotics, all cause substantial modifications to the intestinal microbiome. However, despite this extensive interindividual variability, specialists agree on the existence of a global human core microbiota (101, 102).

Commensal bacteria have much shorter generation times than humans and consequently undergo rapid evolutionary changes, adapting quickly to environmental changes. Unfortunately, external forces sometimes shape a microbiome that is detrimental to the host, a state called dysbiosis. Once established, dysbiosis can exert profound effects on the immune system, creating a feedback loop in which host factors and the microbiome (cell components and metabolites) regulate each other, perpetuating the dysbiotic state (103). It is well established that the intestinal microbiota is severely modified during critical illnesses. However, it remains unclear which change occurs first.

The causal mechanisms of dysbiosis in the critically ill are not completely understood, but they likely result from many intrinsic and extrinsic factors, such as widespread antibiotics use, hypoxic injury, inflammation, intestinal dysmotility, epithelial barrier disruption, vasopressors treatment, and sedation (104). The intestinal microbiome of the critically ill differs substantially from that of healthy individuals and is characterized by lower phylogenetic diversity, commensal microbe loss, and pathobiont overgrowth (105, 106).

## Concluding Remarks

Older adults in critical care conditions develop a peculiar inflammatory response, which is associated with poorer outcomes. Current treatments are unspecific and mainly rely on life support techniques. Novel strategies are under investigation (99), and Personalized Medicine has been widely discussed to improve care of the critically ill (107); however, to a large extend, these proposals still remain experimental and hypothetical, without impacting clinical applications. Thus, manipulation of the inflammatory storms that are so frequent in Critical Care remains a challenging task, filled with negative results and nebulous findings. Prolonged hospital stays, recurrent infections, decrepitude, and malnutrition characterize the critically ill population as a whole, but particularly apply to the subset composed of aged adults.

By the other hand, impressive advances in the molecular biology of aging are emerging. Biomarkers of aging have been extensively investigated to guide tailored treatments of the aging process, as well as to detect individuals that age faster (108). Despite the current challenges (109–111), *in vivo* partial cellular reprogramming (112), direct reprogramming (109), and epigenetic interventions (67) are tentative highways for drug development and captivating platforms to reach this goal. Indeed, the rapid advancement of scientific knowledge in this field provides hope that, in a not-so-distant future, sophisticated medical technologies to delay and even reverse normal aging might be available.

## Author Contributions

FPS conceived and wrote the manuscript. MCCM contributed with suggestions and ideas.

## Conflict of Interest Statement

The authors declare that the research was conducted in the absence of any commercial or financial relationships that could be construed as a potential conflict of interest.
